# An Antidote to Decreasing Interest in Radiation Oncology: Earlier Engagement

**DOI:** 10.1007/s13187-024-02478-x

**Published:** 2024-07-20

**Authors:** Catherine Sport, Nophar Yarden, Emma C. Fields

**Affiliations:** 1 Virginia Commonwealth University School of Medicine, 1201 E Marshall Street, Richmond, VA 23298 USA; 2 Department of Radiation Oncology, Virginia Commonwealth University, Richmond, VA 23219 USA

## Abstract

**Purpose:**

In recent years, there has been a national decline in applicants to radiation oncology (RO) residencies, partly due to limited exposure to RO during medical school. Student Interest Groups (SIGs) give students early exposure to a variety of specialties. This study investigates the efficacy of a RO-SIG to increase knowledge and interest in the field.

**Methodology:**

First and second-year medical students attending an RO-SIG event or shadowing experience completed surveys both prior and following participation. Students ranked their interest in oncology, in RO, and their perceived accessibility of mentors in oncology. Questions were rated on a Likert scale from 0 to 5 (5 highest, 0 lowest). The survey included one short response question about the understanding of the role of the RO, which was evaluated qualitatively.

**Results:**

44 students (42 M1s, 2 M2s) completed the pre-survey and 18 (41%, 17 M1s, 1 M2) completed the post-survey. Of the 18 matched responses, interest in oncology increased from 3.67 pre-SIG to 3.89 (*p* = 0.19) and in RO specifically from 3.17 to 3.89 (*p* < 0.01). The mean perceived accessibility of faculty mentors in oncology increased from 3.18 to 3.72 (*p* < 0.01). After interacting with the RO-SIG, the short response answers were more detailed in the understanding of the role of RO.

**Conclusions:**

RO-SIGs can increase interest in RO through early exposure to the field. In a time where RO has seen a decline in student interest, RO-SIGs are an option to increase engagement, develop interest, and form relationships with mentors in pre-clinical years.

## Introduction

There has been a national decline in the number of applications to radiation oncology (RO) residencies in recent years [1]. Until 2018, the number of applicants into a post-graduate 2nd-year (PGY2) radiation oncology program had been steady at around 190 allopathic residents per year [2]. In 2019, there was an abrupt decrease in the number of applicants to 163 allopathic residents [2]. From 2018 to 2023, the number of applicants decreased by approximately 40%, from 194 allopathic applicants to 119 [2].

The decline in applications has been attributed to several factors, including concerns about residency expansion, restrictions in locations of practice, demanding licensing processes, and a heavy focus on research [3, 4]. Another concern is that most medical students have limited to no exposure to the field during their preclinical or clinical years. In a large survey of medical students in 2021, the majority of students (60.8%) reported no exposure to radiation oncology during medical school. Of those who did have exposure to radiation oncology, about 30% were introduced through the medical school curriculum or a lecture, but < 10% had an opportunity to participate in a rotation [3].In a 2021 study, potential applicants reported significant concerns regarding limited or no exposure to the field during medical school [3].

Student Interest Groups (SIGs) offer students early exposure to a variety of specialties during medical school. Oncology-related SIGs have been shown to increase interest in oncology-related fields and assist students in making career decisions [5, 6]. At our institution, the radiation oncology SIG was separated from the oncology SIG and designed to encourage more students to have exposure to the field of radiation oncology, specifically.

The primary goals of our study were to determine if interaction with events hosted by the radiation oncology SIG increased interest and knowledge about the field in first and second-year medical students.

## Methods

This prospective cohort study included first and second-year medical students who participated in the radiation oncology SIG from 2022 to 2023. During the 2022–2023 academic year, there were 3 SIG events. Additionally, one-day shadowing opportunities were offered to all first and second-year medical students throughout the year.

SIG events included an informational session and presentation, a career panel, and a mock tumor board. The shadowing opportunities were offered to all first and second-year students via e-mail. When shadowing, students spent one day with a radiation oncologist in the university’s department.

Students received a survey when registering for an event hosted by our SIG or for shadowing. This survey included 3 Likert-scale questions and one free response. Follow-up surveys with the same questions were then sent to students again following their interaction with the field. The Likert questions on the survey included asking the students to rate from 0 to 5 (5 highest, 0 lowest) their interest in oncology, interest in radiation oncology specifically, and their perceived accessibility of mentors in oncology fields. The additional short response question was, “What is your understanding of the role of radiation oncology?” Analysis of the quantitative data was completed by matching responses from the initial and follow-up surveys and compared with t-tests. Responses were matched using student identifiers, including name and email. Answers to the free-text questions were analyzed qualitatively based on themes generated by responses.

We collected this data between 2022 and 2023. This study was approved by the medical university’s Institutional Review Board.

## Results

In total, 44 students (42 M1s, 2 M2s) completed the pre-SIG survey and 18 (41%, 17 M1s, 1 M2) completed the follow-up survey after attending a RO-SIG event (12 after specialty panel, 4 after shadowing, 2 after mock tumor board). Mean survey results for all participants and matched participants are shown in Tables 1 and 2, respectively.

Of the 44 pre-survey responses, the interest in oncology was 3.61 out of 5. The interest in radiation oncology specifically was 3.23. The perceived accessibility of mentors in the field was 2.91. There were 12/44 (27.3%) of students who said they did not know anything about radiation oncology, 22/44 (50%) endorsed a basic understanding that we can “use radiation to treat cancers”, and 10/44 (22.7%) students noted how radiation oncologists “work as part of a team” to deliver treatment to patients.

**Fig. 1:**
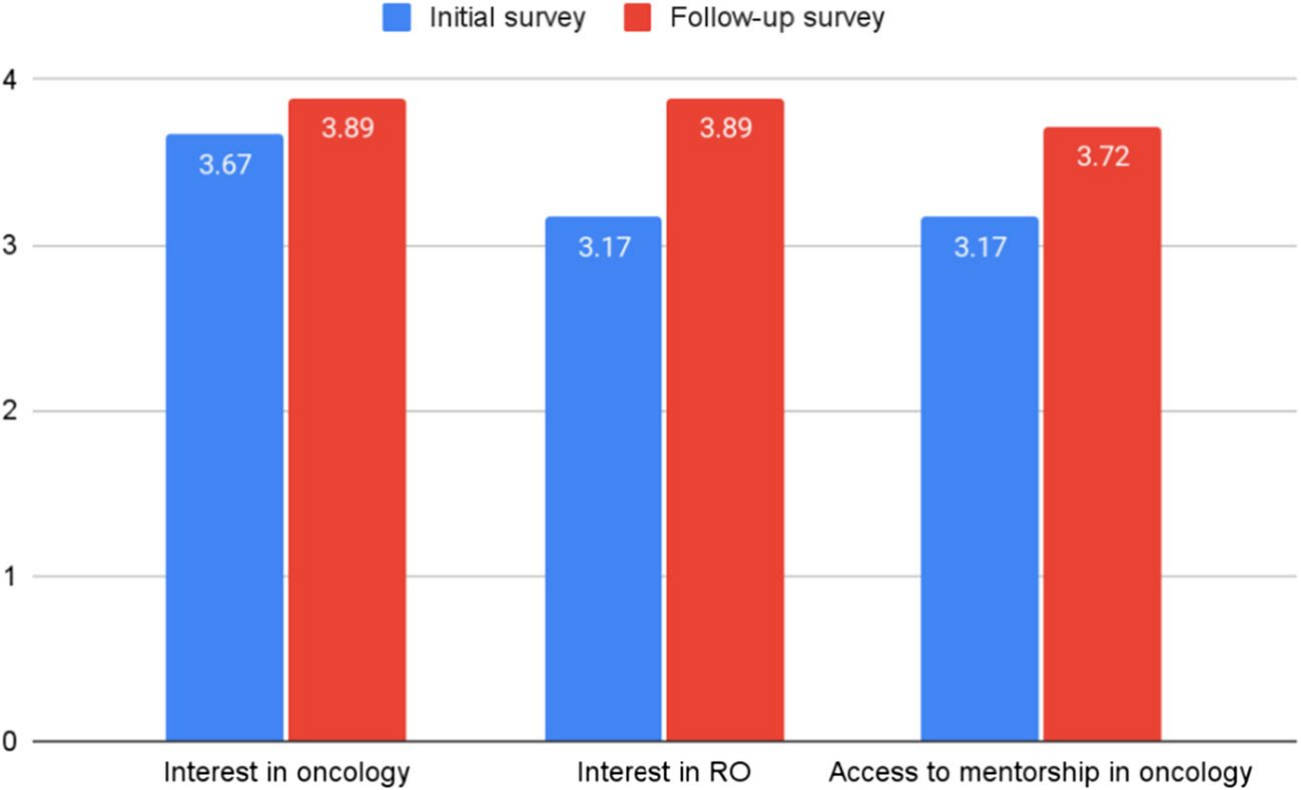
Matched mean survey responses completed prior-to and after participating in an RO-SIG organized experience (*n* = 18)

In assessing the 18 matched responses as shown in Fig. 1, the interest in oncology increased from 3.67 pre-SIG events to 3.89 (*p* = 0.19). The interest in radiation oncology specifically increased from 3.17 to 3.89 (*p* < 0.01). The mean perceived accessibility of faculty mentors in oncology rose from 3.17 to 3.72 (*p* = 0.048).

Of the matched responses, before interacting with the RO-SIG, 4/18 (22.2%) of students endorsed a minimal knowledge of radiation oncology, 10/18 (55.6%) said radiation oncologists “use radiation to treat cancers,” and 4/18 (22.2%) said that radiation oncologists “work in a team of oncologists.” After the event, answers were more detailed in the understanding of the radiation oncologists’ ‘long-term relationships with patients’’ and “ability to both cure and palliate cancer patients.”

## Discussion

This is the first study specifically evaluating the role of a Radiation Oncology Student Interest Group (RO-SIG) in increasing interest in radiation oncology for pre-clinical medical students. This study found that prior to participation in RO-SIG events, the majority of student group members had only a minimal to basic understanding of radiation oncology. This highlights the lack of teaching about the field of radiation oncology during the preclinical years. Participation in RO-SIG events increased participants’s understanding of the specialty. In addition to teaching students more about the field, our study found that participation in RO-SIG events had the positive effect of increasing interest in oncology and radiation oncology, specifically. On the part of RO residents and faculty, participation increased students’ perception of the accessibility of mentors in the field.

Previous studies investigating the effect of early exposure to radiation oncology through a preclinical elective program have also found that exposure to the field increased interest in radiation oncology [7, 8]. In a time where there has been a noted decrease in residency applicants to radiation oncology, RO-SIGs and related activities that promote early exposure to the field may serve as a way to counteract this decline. This study assessed the efficacy of an RO-SIG which organized a specialty panel, shadowing experiences, and a mock tumor board. Prior studies have assessed the effect of introducing students to radiation oncology through similar activities. One study found that medical student shadowing during tumor boards significantly increased interest in both radiation oncology as well as improved understanding of the field [9]. Another study sought to expose medical students to the field through a Pre-Clerkship Residency Exploration Program, which was an elective that allowed students to rotate through the radiation oncology department [10]. Participants endorsed a growth in their perception of radiation oncology in a variety of areas including their understanding of the patient population, career outlook, and research opportunities. Participants also noted they were able to learn more about radiation oncology from residents; prior to this experience, students did not rank learning from residents as a significant source of information. A separate study explored the efficacy of starting a structured didactic program for students participating in a radiation oncology clerkship [11]. Students responded positively to this curriculum, noting that it increased their understanding of the specialty and helped with their specialty decision-making. Overall, this study and previous studies at other institutions demonstrate that medical students respond positively to early exposure to radiation oncology and are likely to have an increased understanding of the specialty through these early hands-on experiences.

In this study, perceived accessibility to mentorship in the field increased after participation in RO-SIG events. Mentorship in radiation oncology has been a subject of previous studies as well. Mentorship programs have been received positively by participants, with mentees noting that such programs have increased their confidence in accomplishing career goals [12]. Participation in these programs also provided mentees with additional research exposure and opportunities to publish research [12–14]. One study found that 29.3% of participants from a radiation oncology mentorship program went on to apply into the specialty [14]. A study completed at Boston University showed that of students that successfully matched into radiation oncology between 2005 and 2020, approximately 81% participated in a structured mentorship program [15]. Overall, medical students respond positively to the opportunity to learn from mentors in the field and it is likely linked to increased interest in the field. Objective measures of research productivity and future application into the specialty are also increased by mentorship programs.

Limitations of this study are that it is a single-institution study with a small sample size. Additionally, not all respondents to the pre-survey completed the post-survey. Future studies could incorporate surveying students from multiple institutions. As we continue to develop our shadowing program, it will be interesting to compare whether there is a difference in student engagement following participation in a career panel compared to spending time in a clinic shadowing. It would also be interesting to have a follow-up study of survey respondents to determine if they ultimately applied to radiation oncology.

The RO-SIG provides further evidence of the value in providing medical students with early exposure to radiation oncology. Students also respond positively to mentorship in the field. In the future, it would be beneficial to explore the success of piloting a mentorship program that pairs attendings and residents with members of the RO-SIG. This study specifically demonstrates the efficacy of student interest groups and proposes events that have been well-received by students, which other institutions could apply as a starting point in piloting a student interest group for their student body. As a specialty that has experienced a decline in applicants in recent years, this study highlights how early exposure to the field could serve as an antidote to this trend.
